# COVID-19, Green Deal and recovery plan permanently change emissions and prices in EU ETS Phase IV

**DOI:** 10.1038/s41467-022-28398-2

**Published:** 2022-03-04

**Authors:** Kenneth Bruninx, Marten Ovaere

**Affiliations:** 1 Department of Mechanical Engineering, Faculty of Engineering Science, KU Leuven, Leuven, Belgium; 2 EnergyVille, Genk, Belgium; 3 Department of Economics, Faculty of Economics and Business Administration, Ghent University, Ghent, Belgium; 4 School of the Environment, Yale University, New Haven, CT USA

**Keywords:** Climate-change mitigation, Climate-change policy, Environmental economics, Energy economics, Energy policy

## Abstract

The EU emissions trading system’s (ETS) invalidation rule implies that shocks and overlapping policies can change cumulative carbon emissions. This paper explains these mechanisms and simulates the effect of COVID-19, the European Green Deal, and the recovery stimulus package on cumulative EU ETS emissions and allowance prices. Our results indicate that the negative demand shock of the pandemic should have a limited effect on allowance prices and rather translates into lower cumulative carbon emissions. Aligning EU ETS with the 2030 reduction target of −55% might increase allowance prices to 45–94 €/ton CO_2_ today and reduce cumulative carbon emissions to 14.2–18.3 GtCO_2_ compared to 23.5–33.1 GtCO_2_ under a −40% 2030 reduction target. Our results crucially depend on when the waterbed will be sealed again, which is an endogenous market outcome, driven by the EU ETS design, shocks and overlapping climate policies such as the recovery plan.

## Abstract (web summary)

This paper finds that the EU’s 2030 reduction target of -55% might correspond to EU ETS allowance prices between 45 and 94 e/ton CO2 today, while the invalidation rule reduces carbon emissions to 14.2 to 18.3 GtCO2 over the EU ETS’ remaining lifetime.

## Introduction

The European Union Emissions Trading System (EU ETS), a cornerstone of EU climate policy, is being put to the test by three large shocks affecting the demand for or supply of emission allowances: a temporary negative allowance demand shock because COVID-19 lockdowns reduced energy demand^1–3^, a positive or negative allowance demand change because of overlapping policies from the NextGenerationEU recovery stimulus package, and a permanent negative allowance supply adjustment because of the EU’s more ambitious EU ETS emissions reduction target of 61% by 2030 compared to 2005 levels^4^, as part of the proposed Fit for 55 Package, implementing the goals of the European Green Deal^5^.

Under an emissions trading system with a fixed cap, these shocks would only affect the price of carbon emissions, not cumulative emissions, which would continue to equal the cumulative emissions cap over the lifetime of the EU ETS—the so-called waterbed effect, as you can push down on a waterbed in any location, but the total volume of water in the bed remains the same^6^. This situation prevailed in the EU ETS during the 2009 recession, which may have contributed to the decreased allowance prices for almost a decade^7^.

In 2018, however, the EU strengthened the EU ETS by adding an invalidation rule to its market stability reserve (MSR) to address the large surplus of allowances in the system and to structurally signal future scarcity of emission allowances^8^. If the number of allowances in circulation (TNAC) surpasses 833 MtCO_2_, the MSR absorbs a share of the allowances to be auctioned, so that they can be released again from the reserve in the future when the TNAC drops below 400 MtCO_2_. Starting in 2023, an invalidation rule will be in effect, such that allowances held in the MSR exceeding the amount auctioned during the previous year will be invalidated^8^. In its Fit for 55 Package, the European Commission proposes to maintain the TNAC thresholds and this invalidation rule, but to fix the invalidation threshold to 400 MtCO_2_ in order to enhance the predictability of the holdings of the MSR^4^. In addition, if the TNAC falls between 833 MtCO_2_ and 1096 MtCO_2_, the difference between the TNAC and 833 MtCO_2_ is placed in the MSR to avoid threshold effects^4^.

Because the EU ETS’ invalidation rule is conditional on the TNAC^8^, changes to allowance demand will affect the number of invalidated allowances and hence change cumulative carbon emissions. As a result, overlapping policies^6,9–11^ such as the recovery package or the Green Deal, strategies to ‘buy, bank, and burn’ allowances^12^ or exogenous shocks such as the COVID-19 pandemic^1–3^ may result in changes to cumulative emissions. In other words, the waterbed is punctured^6^.

The magnitude and direction of the impact of a policy or shock on cumulative emissions depends on three factors: (i) when the policy affects the demand for emission allowances, (ii) when it is announced, and (iii) the year that the waterbed is sealed. The waterbed seals when the TNAC falls below 833 MtCO_2_ and the MSR stops absorbing allowances. This is an endogenous market outcome that depends on the supply of and the demand for allowances.

First, the timing of a policy or shock affects cumulative emissions because there is a direct effect on the number of allowances that are invalidated. This is not a one-to-one relationship, as the MSR only absorbs a share of the TNAC in a given year. Under the 2018 MSR design^8^, 24% from 2019 till 2023 and 12% from 2024 onward of the TNAC is transferred to the MSR if the TNAC exceeds 833 MtCO_2_. In the Fit for 55 Package, the European Commission proposes (i) to keep the intake rate at 24% until the end of Phase IV (2030) and (ii) to place the difference between the TNAC and 833 MtCO_2_ in the MSR if the TNAC is between 1096 MtCO_2_ and 833 MtCO_2_. As a result, changes in allowance demand before the waterbed is sealed are gradually transferred to the MSR and allowances invalidated. Actions taking place after the waterbed has been sealed again will not have a direct effect.

Second, the announcement of a policy matters because expectations about future changes to emission allowance demand might affect behavior today. This indirect effect happens through adjustments of the emission allowance price profile^10,11,13^. For example, if one announces today that a coal plant will close in the future, market participants expect the future price of allowances to drop, as the total number of allowances in circulation will increase in the future. Because EU ETS allowances are bankable and have an infinite lifetime, the future drop in allowance prices will lead to lower prices today, assuming market participants are intertemporally optimizing. As a result, the incentive to abate today will decrease because of expected carbon abatement in the future, decreasing the TNAC and invalidation volumes early on. Similarly, announced future decreases of the TNAC will lead to higher emission allowance prices, higher abatement and a higher TNAC today, which in turn results in more invalidation. The indirect price effect persists as long as the policy affects the demand for emission allowances in a period that the TNAC is not zero, hence, firms are still intertemporally optimizing or banking. It may work in the opposite direction of the policy or shock, i.e., a policy reducing (increasing) the demand for emission allowances may increase (decrease) cumulative emissions over the lifetime of EU ETS^10,11,13^. The mechanism for such a backfiring policy is similar to the Green Paradox in intertemporal carbon leakage (shifts in carbon emissions between time periods)^14^ and spatial carbon leakage (the effort of abating countries may be offset by increasing emissions in non-abating countries)^15^. Note, however, that the literature on carbon leakage focuses on carbon abatement, while we consider both policies that decrease and policies that increase the demand for emission allowances. This can lead to both lower and higher emissions from within the waterbed, so the aggregate effect can go in the opposite direction of intertemporal or spatial carbon leakage^16–18^.

Third, as the year in which the waterbed seals is an endogenous market outcome, all changes to the TNAC can potentially affect when the TNAC falls below 833 MtCO_2_. In addition to shocks and policies that directly affect the TNAC today, the TNAC also changes due to today’s expectations about future abatement actions. Generally, any measure that increases perceived abatement costs in the future, encourages banking of allowances now, which may prolong the duration of the waterbed puncture, because the TNAC increases and vice versa (see Methods for a more extensive discussion and examples of this mechanism)^19^.

**Fig. 1:**
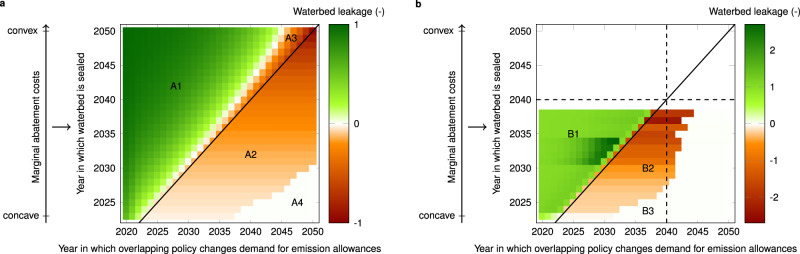
Waterbed leakage (the net effect of a change in allowance demand on cumulative emissions in EU ETS) as a function of the year the overlapping policy or shock takes place (horizontal axis) and the year the waterbed is sealed (vertical axis). **a** Assumes a 40% emission reduction target by 2030 (2018 EU ETS and MSR design), whereas in **b** this target is increased to −55% (Fit for 55 EU ETS and MSR design). In **b**, the increased linear reduction factor of 4.2% (proposed by the European Commission in its Fit for 55 Package^4^) is kept constant after 2030, hence, the cap equals zero in 2040 (dashed lines). The solid black diagonal line indicates when the direct effect ends, i.e., a change in emission allowance demand in any year after the waterbed is sealed does not entail a direct effect. Waterbed leakage is positive when a policy that increases (decreases) the demand for emission allowances in a particular year leads to increased (decreased) cumulative emissions. It is negative when a policy backfires. The timing of sealing can be varied by adding shocks or overlapping policies (Fig. 2), or by varying the convexity of the marginal abatement cost curve (see Methods), as indicated by the arrow on the left (in the figure). The numerical values behind these graphs are reported in the source data, while a separate graphical representation of the direct and indirect effect can be found in the Supplementary material.

In this work, we provide numerical estimates of the impact of overlapping policies and shocks on cumulative emissions over the lifetime of EU ETS, which we summarize as waterbed leakage, depending on the year in which the shock or overlapping policy changes the demand for emission allowances and the year in which the waterbed is sealed again. We do this considering the 2018 EU ETS and MSR design^8^ as well as the proposed EU ETS and MSR design in the Fit for 55 Package^4^, highlighting the implications of the proposed design changes on the impact of overlapping policies on cumulative emissions. For shocks and overlapping policies “on the margin”, i.e., that do not affect the duration of the waterbed puncture, we find that the direct effect may affect cumulative emissions more than proportionally under the EU ETS design proposed in the Fit for 55 Package due to an interaction between the changes to the MSR intake rate, the TNAC definition and the timing of the MSR’s supply adjustments (see Fig. 1b and Methods). For pre-announced policies that change allowance demand before the waterbed seals, these more-than-proportional supply adjustments trigger indirect effects in the same direction as the emission allowance demand adjustment induced by the policy or shock, further amplifying the direct supply adjustments. Note that this is opposite to what we find for the 2018 MSR design, where the indirect effect always works in the opposite direction as the direct effect. Moreover, the more-than-proportional direct effect amplifies the indirect effect for pre-announced policies that change allowance demand after the waterbed seals, resulting in more-than-proportional changes in cumulative emissions in response to backfiring policies. Although theoretically possible^20^, we do not find such more-than-proportional supply adjustments under the 2018 MSR design. This analysis allows policymakers and market actors to operationalize these concepts in their assessment of shocks or overlapping policies “on the margin”. In addition, we quantify the individual and joint effect of the COVID-19 shock, the European Green deal, and the recovery stimulus package on allowance prices and cumulative emissions. These major disruptions fundamentally change the equilibrium between demand and supply of emission allowances, changing the duration of the waterbed puncture. Our results indicate that the negative demand shock of the pandemic should have a limited effect on the EU ETS price and rather translates almost into lower cumulative carbon emissions. Aligning EU ETS with the 2030 reduction target of −55% might increase allowance prices to 45–94 €/ton CO_2_ today and reduce cumulative carbon emissions to 14.2–18.3 GtCO_2_ compared to 23.5–33.1 GtCO_2_ considering the 2018 EU ETS and MSR design^8^. Our results crucially depend on when the waterbed will be sealed again, which is an endogenous market outcome, driven by the EU ETS design, shocks, and overlapping climate policies such as the recovery plan.

## Results

### Waterbed leakage: impact of overlapping policies “on the margin”

Because of the punctured waterbed, the effect of overlapping policies on cumulative emissions is not obvious and depends on the year the policies are announced, the time profile of their effect on the demand for emission allowances, and the year in which the waterbed is sealed^10–13,21^. We summarize the effect of any change to allowance demand on cumulative emissions as waterbed leakage:

1 $$\,{{\mbox{Waterbed leakage}}}\,=\frac{{{\Delta }}\ \,{{\mbox{cumulative EU ETS emissions}}}\,}{{{\Delta }}\ \,{{\mbox{allowance demand}}}\,}$$

which is defined as the final change in cumulative emissions over the lifetime of the EU ETS (numerator), caused by a positive or negative change to allowance demand (denominator). Waterbed leakage is therefore positive when a policy that changes the demand for allowances leads to a change in cumulative emissions in the same direction. For example, waterbed leakage is positive if a coal phaseout (which decreases the demand for emission allowances by displacing high-carbon coal by less carbon-intensive alternatives such as natural gas and renewables) decreases cumulative EU ETS emissions. Waterbed leakage is also positive if a nuclear phaseout (which increases the demand for emission allowances by displacing zero-carbon nuclear by more carbon-intensive alternatives such as natural gas) increases cumulative EU ETS emissions. If the cap is fixed and there is no allowance invalidation, a policy will have no effect on cumulative emissions and waterbed leakage equals zero. If a policy’s effect on cumulative emissions is opposite to its change in allowance demand, waterbed leakage is negative.

**Fig. 2:**
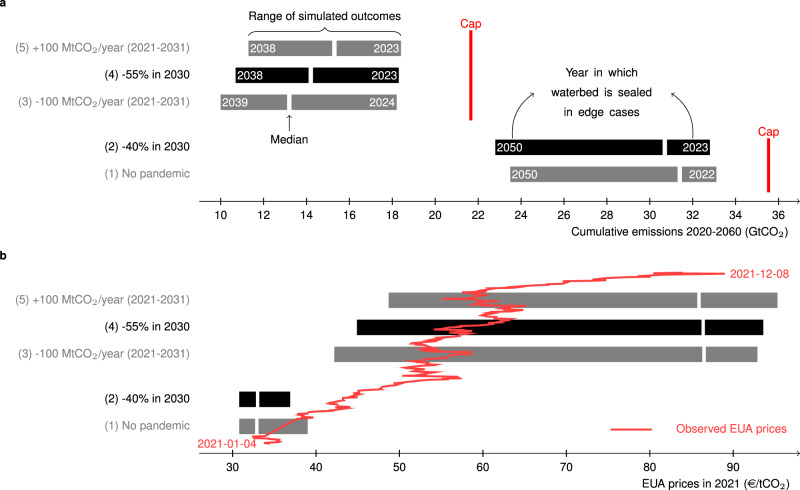
Cumulative emissions and allowances prices in 2021 in each of our five scenarios. The black and gray bars represent the range of cumulative emissions (**a**) and allowances prices in 2021 (**b**), the white markers represent the results under reference assumptions, which lead to a sealed waterbed in 2030 in the (1) “No pandemic scenario”, and the vertical red lines represent the cumulative cap without invalidation (2020-end ETS). In **a**, he indicated years are the years in which the waterbed is sealed in the edge cases (lowest-highest cumulative emissions). In **b**, the solid red line indicates observed emission allowance prices between January 4, 2021 and December 10, 2021. The cumulative impact of the COVID-19 pandemic is assumed to be 0.72 GtCO_2_ in the period 2020–2025 and is enforced in all scenarios except the “No pandemic” scenario. Scenario (3) and (5) mimic the effect of an overlapping policy reducing (3) or increasing (5) the demand for emission allowances by 100 MtCO_2_/year in the period 2021–2030, considering the −55% emission reduction target.

Figure 1 presents a graphical summary of waterbed leakage depending on two of the above three dimensions: the year in which the overlapping policy reduces the demand for emission allowances on the horizontal axis and the year in which the waterbed is sealed on the vertical axis—assuming that the policy is announced in 2020. Panel (a) assumes a 40% emission reduction target by 2030 and the 2018 MSR design^8^, whereas in panel (b) this target is increased to −55% and the design of the MSR has been adjusted, in line with the proposed changes under the Fit for 55 Package^4^. The set of equilibria underpinning this analysis was obtained as follows. First, we create a set of scenarios in which the waterbed seals sooner or later, one for each year between 2023 and 2050 under the 2018 EU ETS and MSR design (Fig. 1a) or 2023 and 2038 under the EU ETS and MSR design in the Fit for 55 Package (Fig. 1b). We do this by varying the convexity of the marginal abatement cost curves that characterize abatement options for the sectors covered by EU ETS. The more convex the marginal abatement cost curve, the later the waterbed seals. Note that varying other parameters (e.g., baseline emissions) or adding different shocks could lead to a similar set of results. Second, we add an overlapping policy in a particular year between 2020 and 2050 to each of the marginal abatement cost curves above by reducing baseline emissions by 1 MtCO_2_. Comparing cumulative emissions before and after adding the overlapping policy allows calculating waterbed leakage. For a more detailed discussion, see Methods. Importantly, we only consider marginal policies that do not affect the duration of the punctured waterbed or the year in which the TNAC drops below 833 MtCO_2_ or 1096 MtCO_2_, see Fig. 2, as such policies could lead to waterbed leakage that can have any positive or negative value. Hence, in Fig. 1a displaying waterbed leakage under the 2018 EU ETS and MSR design, waterbed leakage is always below 1^20^. In contrast, waterbed leakage can be above 1 considering the Fit for 55 EU ETS and MSR design (Fig. 1b).

We can identify different regions in Fig. 1, based on the relative importance of the direct effect of the policy on cumulative emissions^6^ and the indirect price effect caused by the adjustment of the equilibrium price^11^ (see Supplementary Figs. 1 and 2 for a detailed representation of the direct and indirect effect separately).

First, in the upper-left part of the figures, the direct effect dominates, because the overlapping policy is executed well before the waterbed is sealed. In that case, changes to the TNAC affect invalidation over an extended period. Under the 2018 EU ETS and MSR design, the most extreme case occurs if one executes an overlapping policy in 2020 and the waterbed seals in 2050 (Fig. 1a, A1), as explained by Perino^6^: direct waterbed leakage nearly equals 1. In other words, a decrease in allowance demand now decreases cumulative emissions by nearly the same amount. On the other hand, the closer the effect of the overlapping policy is to the year in which the waterbed is sealed, the lower the direct effect. Under the 2018 MSR design^8^, the direct effect is always positive and converges to 1 as (i) the change in allowance demand takes place earlier and (ii) the waterbed is sealed later.

Under the proposed design for EU ETS and the MSR in the Fit for 55 Package, direct waterbed leakage, however, may equal or exceed 1 for policies executed before the waterbed seals due to an interaction between the changed intake rates of the MSR, the TNAC definition and the timing of the MSR’s supply adjustments (Fig. 1b, B1). This happens if the TNAC is in the range 833 MtCO_2_ to 1096 MtCO_2_ for at least one but less than four consecutive years after the overlapping policy reduces emission allowance demand (see Methods for a discussion on these conditions). In our simulations, this occurs in all cases in Fig. 1b except when the waterbed seals in 2023. The impact of a policy executed in the year before the waterbed seals on the TNAC is entirely absorbed and invalidated by the MSR, as the intake in that year equals the difference between the TNAC and 833 MtCO_2_. Direct waterbed leakage for these policies, hence, equals 1. If a policy is executed 2 or more years before the waterbed seals, direct waterbed leakage exceeds 1. As the MSR affects the supply of allowances proportionally between September and August, whereas the TNAC is calculated at the end of December, only 1/3 of the supply change is accounted for in the calculation of the TNAC at the end of the following year (see “Methods” for a numerical example). This leads to a more than proportional increase in intake, hence, invalidation, in response to overlapping policies. The direct effect peaks at 1.67 when (i) the policy is executed 2 years before the waterbed is sealed again and (ii) the TNAC is in the range 833 MtCO_2_ to 1096 MtCO_2_ in those 2 years, which occurs when the waterbed seals between 2031 and 2034 in Fig. 1b.

Second, below the diagonal line, the direct effect is zero as the overlapping policy only reduces the demand for emission allowances after the waterbed is sealed again. However, waterbed leakage is not zero, because of the indirect effect of announced future overlapping policies on the equilibrium price path, which affects the demand for emission allowances before the waterbed is sealed. This indirect price effect is negative for policies affecting emission allowance demand after the waterbed is sealed again, meaning that overlapping policies backfire: abatement efforts lead to an increase in cumulative emissions and announced increases in the demand for emission allowances lead to a decrease in cumulative emissions (Fig. 1a, A2 and Fig. 1b, B2). This ‘new green paradox’ was first described by Rosendahl^11^. In general, for a given duration of the waterbed puncture, the indirect effect increases with the time between the announcement of a policy and when it takes place^13^. But as soon as the waterbed is sealed, the indirect effect becomes independent of the year in which the policy is executed^13^. The indirect price effect is larger when (i) the waterbed is sealed later; (ii) the policy is announced today, but executed later and (iii) the period over which firms bank allowances after the waterbed is sealed is shorter. As a result of (i) and (ii), waterbed leakage is more negative in the upper-right corner of Fig. 1a (A2) and Fig. 1b (B2). Under the 2018 MSR design, the indirect effect is always negative and may dominate the direct effect in the years preceding the year in which the waterbed is sealed, yielding negative waterbed leakage in regions where one intuitively expects positive values (Fig. 1a, A3). Although theoretically possible under the 2018 MSR design^20^, we do not observe waterbed leakage below −1 in our simulations.

Under the Fit for 55 MSR design, we find positive indirect effects—i.e., in the same direction as the direct effect, resulting in waterbed leakage values up to 2.71—for pre-announced policies affecting emission allowance demand before the waterbed seals (Fig. 1b, B1) and negative indirect effect values up to −2.36 for pre-announced policies affecting emission allowance demand after the waterbed seals (Fig. 1b, B2). Both are the result of the more-than-proportional supply adjustment in the Fit for 55 MSR design. For policies that affect emission allowance demand before the waterbed seals, the potentially more-than-proportional supply adjustment triggers price adjustments in the opposite direction than one would intuitively expect from the overlapping policy (i.e., a reduction in emission allowance demand increases emission allowance prices and vice versa), amplifying the effect of the overlapping policy. After the waterbed seals, the backfiring indirect effect of pre-announced policies and the resulting changes in emission allowance demand may translate in supply adjustments reinforced by the direct effect, leading to more negative waterbed leakage compared to the values observed under the 2018 MSR design.

Third, as soon as there are no more banked allowances and the total number of allowances in circulation is zero, there is no more intertemporal arbitrage and the EU ETS once again puts a strict cap on cumulative emissions (Figs. 1a, A4 and b, B3). In these years banking is zero, overlapping policies only affect emission allowance prices, but not cumulative emissions, and there could have been borrowing if this was allowed (which it is not in EU ETS). The sooner the waterbed is sealed, the sooner the bank will be zero. As both the direct effect and the indirect price effect are zero, waterbed leakage is zero. Overlapping policies will not have any effect on cumulative emissions but will affect the emission allowance price.

So far, we focused on policies or shocks that affect allowance demand. A change in allowance supply might have a symmetric, opposite waterbed leakage, meaning that the waterbed leakage of a positive (negative) allowance demand shock in a specific year is equal to the waterbed leakage of a negative (positive) allowance supply shock in the same year. This equivalence only holds if the changed supply does not change the invalidation threshold. As the invalidation threshold is constant and equal to 400 MtCO_2_ in the Fit for 55 Proposal^4^, Fig. 1b can also be used to quantify the impact of supply shocks on the margin, i.e., that do not affect the duration of the waterbed puncture. Under the 2018 EU ETS and MSR design (Fig. 1a), this is only the case if the supply shock does not alter the auction volumes.

### The impact of COVID-19, the Green Deal, and the recovery plan

Figure 2 summarizes the simulated (a) cumulative emissions and (b) allowances prices for five policy scenarios, which are constructed as follows. Scenario (1) and (2) consider the −40% carbon reduction target by 2030, whereas scenarios (3–5) consider a reduction target of −55%, reflecting the implementation of the Green Deal. For ease of referencing, we characterize our scenarios based on the overall European emission reduction target (−40% or −55%) relative to 1990 emission levels. Recall, however, that the emission reduction targets for the sectors covered by EU ETS are more stringent: −43% (2018 EU ETS and MSR design^8^) or −61% (proposed by the European Commission in its Fit for 55 Package^4^), relative to 2005 emission levels. This results in linear reduction factors of 2.2% and 4.2% as of 2021, kept constant until the emissions cap equals zero^4^. In both cases, we rebase the overall emissions cap in 2021 to reflect the impact of Brexit on EU ETS^22^ and include aviation in the European Economic Area^8^. In scenarios (3) to (5), we include maritime transport as of 2021, whereas the European Commission proposes to do so after 2023^4^. Note furthermore that the Fit for 55 Package will enter into force after 2021, which will require a one-off emission allowance supply reduction to ensure the linear reduction factor equals 4.2% in Phase IV. The impact of the timing of this one-off reduction in allowance supply is not studied in this paper.

Scenario (1) does not consider the impact of COVID-19 (reducing baseline emissions by 0.72 GtCO_2_ in the period 2020–2025, the worst-case estimate in Bruninx and Ovaere^2^), whereas all other scenarios do. Scenario (3) and (5) mimic the effect of an overlapping policy—induced by the recovery stimulus package—affecting emission allowance demand by 100 MtCO_2_/year in the period 2021–2030, considering the −55% emission reduction target. In scenario (3), emission allowance demand decreases by 100 MtCO_2_/year, as the result of, e.g., support for renewable generation, which displaces fossil fuel-based electricity generation. In scenario (5), emission allowance demand increases by the same amount, as the result of, e.g., an accelerated uptake of electric vehicles and heat pumps, because gasoline or natural gas (currently not covered by EU ETS) is displaced by electricity (covered by EU ETS). See Methods for a detailed description of all five scenarios. The key differences between the scenarios are summarized in Table 1.

**Table 1:** The five considered policy scenarios differ w.r.t. the emissions cap as of 2021, the linear reduction factor (LRF) after 2021, the intake rate of the MSR in the period 2024–2030, the inclusion of the negative demand shock because of the COVID-19 pandemic, and the inclusion of an additional negative or positive demand shock in 2021–2030.

Policy scenario	Cap	LRF	MSR intake	Pandemic	Additional
	2021	after 2021	rate 2024–2030	shock	shock
	(MtCO_2_)	(%)	(MtCO_2_)	(–)	(–)
(1) No pandemic	1596	2.2%	12% TNAC if TNAC≥833	✗	✗
(2) −40% in 2030				✓	✗
(3) −100 MtCO_2_/year (2021–2030)	1635	4.2%	$$\left\{\begin{array}{l}{{{{{{{\rm{TNAC}}}}}}}}-833\,{{{{{{{\rm{if}}}}}}}}\,833\,\le \,{{{{{{{\rm{TNAC}}}}}}}}\,\le \,1096\\ 24 \% \,{{{{{{{\rm{TNAC}}}}}}}}\,{{{{{{{\rm{if}}}}}}}}\,{{{{{{{\rm{TNAC}}}}}}}}\, > 1096\end{array}\right.$$	✓	✓
(4) −55% in 2030				✓	✗
(5) +100 MtCO_2_/year (2021–2030)				✓	✓

The emissions cap in 2021 includes intra-EEA aviation and has been rebased to reflect the impact of Brexit in all scenarios. The emissions cap in scenarios (3)–(5) has been increased to accommodate the inclusion of maritime transport. The MSR intake rate reverts to 12% after 2030 in all scenarios. Additionally, if the TNAC is between 833 MtCO_2_ and 1096 MtCO_2_ after 2030 in scenarios (3)–(5), the difference between the TNAC and 833 MtCO_2_ will be placed in the MSR. Minimum intake rates (200 MtCO_2_ until 2023 and 100 MtCO_2_ thereafter) are only enforced in scenarios (1) and (2).

The vertical red lines in Fig. 2 represent the cumulative cap (2020-end ETS), including the back-loaded and unallocated allowances, as well as the surplus at the end of 2019^5^, in the absence of any invalidation. This means a cumulative cap of 35.5 GtCO_2_ under the 2018 EU ETS and MSR design, in line with a 40% carbon reduction target in 2030 (‘−40% in 2030’). Because the pandemic does not affect the cumulative cap, it is equal under the second scenario (‘−40% in 2030’). When the carbon reduction target in 2030 is increased to −55% (‘−55% in 2030’), the cumulative cap lowers to 21.7 GtCO_2_, despite the extension to maritime transport.

The black and gray bars in Fig. 2a represent simulated cumulative emissions in each of the five scenarios. Because of invalidation, cumulative emissions are always lower than the cumulative emissions cap. In the pre-pandemic scenario (1), we find that cumulative emissions range from 33.1 GtCO_2_ to 23.5 GtCO_2_, depending on when the waterbed is sealed again (indicated by the dates in the bars), which depends on several factors (see Methods). If the waterbed is already sealed in 2022, total invalidation is only 2.4 GtCO_2_, while 12.5 GtCO_2_ of allowances might be invalidated if the waterbed seals in 2050. The white marker represents the results obtained under our reference assumptions, i.e., where the waterbed seals in 2030 in the (1) “No pandemic” scenario. Adding the COVID-induced negative demand shock of 0.72 GtCO_2_ in the period 2020–2025 (scenario (2)), we find that it largely translates into lower cumulative emissions. As discussed above, the longer the duration of the waterbed puncture, the larger the share of the additional 0.72 GtCO_2_ allowances that might be invalidated. Importantly, because the negative demand shock induced by COVID-19 measures increases the number of allowances in circulation, it could prolong the waterbed puncture. For example, in cases where the total number of allowances in circulation is low and the waterbed was expected to be closed in 2022, the negative demand shock of COVID extends the puncture to 2023.

Raising the ambition for 2030 to −55% (scenario (4)), our results indicate that this may lead to more invalidation, especially in the cases with high cumulative emissions (compare scenario (2) and (4)). This is driven by a counter-intuitive self-reinforcing effect^19^: any measure that makes it more costly to meet the emission cap (here: reducing the supply of allowances) incentivizes abatement early-on and banking, which increases the TNAC and, hence, invalidation. Moreover, due to the design of the MSR, this may prolong the duration of the waterbed puncture. In line with our discussion above, we find that the invalidation volume in our reference case might increase with ~2.6 GtCO_2_—from 4.8 GtCO_2_ to 7.5 GtCO_2_. Note that this self-reinforcing effect also explains the variation in emissions within each scenario: concave marginal abatement costs provide less incentive for abatement and banking early-on, hence, lead to less invalidation, whereas strongly convex marginal abatement costs motivate abatement early-on, hence, prolong the waterbed puncture, increase invalidation and lower cumulative emissions (Fig. 1)^19^.

In scenarios (3) and (5), we add a change to the emission allowance demand in scenario (4) of −100 or +100 MtCO_2_/year over the 2021–2030 period. This is an illustration of the effect of an overlapping policy, like renewable support (scenario (3), decreasing allowance demand by displacing fossil-fueled generation) or electric vehicles (scenario (5), increasing allowance demand by displacing non-EU ETS gasoline by EU ETS electricity). In line with the values in Fig. 1, we find that the effect of these policies could be almost completely translated into lower (3) or higher (5) cumulative emissions if the waterbed is punctured for a long time—indicating waterbed leakage close to 1 as the direct effect dominates. Waterbed leakage may be as high as 1.11 (scenario (3)) or 1.22 (scenario (5)) if the waterbed seals in 2033 (scenario (3)) or 2030 (scenario (5)). When the waterbed is sealed soon, the effect on cumulative emissions is, as expected, lower. In these cases, the overlapping policy extends beyond the year in which the waterbed is sealed, such that the indirect effect dominates the direct effect in later years. Furthermore, note that the overlapping policy in scenario (3) changes the duration of the waterbed puncture, once more illustrating that this is an endogenous market outcome.

Zooming in on Fig. 2b, we find that the negative demand shock of the pandemic (scenario (2)) should have a limited effect on the price of allowances in 2021. Since the marginal abatement cost curves are calibrated to reproduce 2019 EUA prices before rebasing the emission allowance cap in 2021 (see Methods) and firms are banking emission allowances beyond 2021 in all cases, variations in 2021 EUA prices in this scenario are limited. The raised ambitions, however, could increase the price in 2021 to a value of 86.5 €/tCO_2_ in our reference case (44.9–93.6 €/tCO_2_). The estimated EUA price is lower when the waterbed is sealed sooner and invalidation is lower. The price changes because of the overlapping policies in scenarios (3) and (5) are larger when the waterbed is sealed sooner because a smaller fraction of the shock is absorbed through invalidation.

## Discussion

Our analysis shows that waterbed leakage—and hence the effect of COVID-19, the Fit for 55 Package, and the recovery plan on the emission allowance price and cumulative emissions—crucially depends on when allowance invalidation stops and the waterbed is sealed again. This is an endogenous market outcome which in itself depends on external shocks (e.g., COVID-19), overlapping policies (e.g., recovery stimulus package), European climate policy, and the shape of the abatement cost curve. This paper does therefore not make predictions of the exact effect of various shocks and policies on emission allowance prices and cumulative emissions in the EU ETS, but it presents a range of possible outcomes and explains the mechanisms driving the punctured waterbed of the EU ETS. The numerical estimates of waterbed leakage allow policy makers and market participants to operationalize this concept in their evaluation of overlapping policies and shocks and highlights the uncertainty on waterbed leakage associated with any policy, as this crucially depends on the year in which the waterbed seals.

Raising the ambition for 2030 to −55%, our analysis finds that allowance prices might increase to 45–94 €/tCO_2_ today and reduce cumulative carbon emissions to 14.2–18.3 GtCO_2_ compared to 23.5–33.1 GtCO_2_ under the 2018 EU ETS and MSR design. Note that EU ETS prices have reached their all-time high in December 2021, above 86 €/tCO_2_, which is within our estimated range and might indicate that the stringency of the new 2030 targets is gradually  internalized by the market. The level of the EU ETS price is important, not only for firms covered by EU ETS and European governments, but also for firms importing goods into Europe because of the proposed border adjustment mechanism, as the level of the carbon border tax in the current proposal is linked to the EU ETS price^23^.

For overlapping policies “on the margin”, the magnitude of waterbed leakage depends on the relative importance of the direct effect of the policy on cumulative emissions and the indirect price effect. In the 2018 MSR design, the direct effect is always less than 1, whereas the Fit for 55 MSR design allows for direct waterbed leakage in excess of 1. Based on an extensive set of numerical simulations, we illustrate, among other things, that all overlapping policies announced now and affecting the demand for emission allowances after the waterbed is expected to be sealed will backfire in absence of proper companion policies (e.g., voluntary cancellation of emission allowances^8^). This, however, requires that the market believes the policy will be executed, observes the corresponding emission allowance price changes and reacts accordingly^24^. In contrast, the direct effect of any policy, regardless of its size or effect on emission allowance prices, will always materialize.

The mechanisms of the current EU ETS design that we illustrate in this paper arise because the supply of allowances depends on the number of allowances in circulation, which makes cumulative emissions endogenous and exacerbates quantity uncertainty. We find that these effects are not mitigated, but reinforced, in the Fit for 55 proposal^4^ and that it proposes to use a similar quantity-based MSR for the separate transportation and heating ETS that will be established in 2026. Perino et al.^20^ propose that allowance supply could be conditioned on the price of allowances instead of the total number of allowances in circulation, similar to the California cap-and-trade system. This has the potential to stabilize prices, but might not sufficiently decrease the uncertainty on cumulative emissions and might lead to oscillatory price behavior between the price cap and floor^25^. This may be an interesting question for future research.

Regarding the Fit for 55 quantity-based MSR design proposal, we offer two suggestions for potential improvements. First, the current threshold at 1096 MtCO_2_ and the 12% intake rate may still induce threshold effects^4^ after 2030. If the TNAC marginally exceeds 1096 MtCO_2_, the intake would equal 131.5 MtCO_2_, whereas if it is marginally below the threshold, intake equals 263 MtCO_2_. This may trigger oscillations in the MSR intake and TNAC, and reduces predictability of the allowance supply. Reducing the threshold after 2030 to 946.5 MtCO_2_ would remove such effects. Second, the waterbed leakage values visualized in Fig. 1b were shown to crucially depend on a direct effect that exceeds 1, which stems from the MSR’s supply adjustments that are currently spread over 2 years. This may be mitigated by frontloading the supply adjustment (i.e., the supply adjustment depending on the TNAC in year *t* − 1 is fully executed in September to December of year *t*, instead of postponing 8/12 to year *t* + 1) or by accounting for future supply adjustments in the calculation of the TNAC (i.e., the TNAC in year *t* − 1 is the difference between cumulative supply and emissions up to year *t* − 1, corrected for supply adjustments in January to August of year *t*). In such a MSR design, the direct effect of any overlapping policy executed before the waterbed seals is less or equal to 1, hence, waterbed leakage always equals 1 if the TNAC falls in the range 833 MtCO_2_ to 1096 MtCO_2_ at least once before the waterbed seals. For overlapping policies that affect emission allowance demand after the waterbed is sealed, waterbed leakage remains negative. Voluntary cancellation may be used to avoid backfiring overlapping climate policies.

Our analysis builds on a deterministic simulation model, assuming perfect foresight and intertemporally optimizing firms, whose abatement options are reflected solely via marginal abatement cost curves. Although such assumptions do not reflect reality, they allow for a first-best analysis, unaffected by imperfections of real-life decision makers. Myopic decision making^26^ may lead to shorter waterbed punctures and lower invalidation volumes. Risk-aversion^27^, on the other hand, may prolong the duration of the waterbed puncture and increase invalidation volumes. Future work may focus on including such myopic decision making under uncertainty, enriching the way abatement options are modeled, e.g., by including more technical and temporal detail^19^, as well as including links with (energy) sectors not covered by EU ETS and the rest of the economy.

## Methods

### Simulation model

We analyze the impact of the three shocks on the emission allowance price and cumulative emissions under EU ETS, using a stylized EU-ETS-MSR model. This model is a simplified version of the detailed long-term investment model of Bruninx et al.^19^ and similar to the one employed by, e.g., Perino and Wilner^28^. This partial equilibrium model assumes a rational, price-taking, and risk-neutral representative firm that optimizes its abatement and banking actions over the complete EU ETS horizon.

In equilibrium, the representative firm abates until its marginal abatement cost (MAC) equals the emission allowance price^28^. Hence, given an emission allowance price path, the representative firm’s emissions are known (Eq. (4)) and the firm minimizes the procurement cost of the required emission allowances to cover these emissions, assuming a discount rate *r*:

2 $$\,{{\mbox{Min.}}}\,\ \ \ \mathop{\sum}\limits_{t\in {{{{{{{\mathcal{T}}}}}}}}}\frac{{p}_{t}\cdot {\tilde{q}}_{t}}{{(1+r)}^{t}}$$

subject to

3 $$\forall t\in {{{{{{{\mathcal{T}}}}}}}}:\quad \mathop{\sum }\limits_{{t}^{* }=1}^{t}{\tilde{q}}_{{t}^{* }}\ge \mathop{\sum }\limits_{{t}^{* }=1}^{t}{q}_{{t}^{* }}$$

4 $$\forall t\in {{{{{{{\mathcal{T}}}}}}}}:\quad {q}_{t}={{{{{{{{\mathcal{F}}}}}}}}}_{t}^{-1}({p}_{t})$$

5 $$\forall t\in {{{{{{{\mathcal{T}}}}}}}}:\quad {\tilde{q}}_{t},{q}_{t}\ge 0$$

The representative firm must buy sufficient allowances ${\tilde{q}}_{t}$ ahead of time to cover its emissions *q*_*t*_ (Eq. (3)). Note that Eq. (3) implies that the representative firm may bank allowances, but that borrowing is not allowed. Equation (4) relates the emissions *q*_*t*_ to the emission allowance price *p*_*t*_ via the marginal abatement cost curve (MACC) ${{{{{{{{\mathcal{F}}}}}}}}}_{t}({p}_{t})$ (see below).

The constraint which enforces the balance between the demand for and supply of emission allowances, and hence, links the decisions of all firms, can be expressed as follows:

6 $$\forall t\,\in \,{{{{{{{\mathcal{T}}}}}}}}:\quad {\overline{q}}_{t}-{\tilde{q}}_{t}\ge 0\quad ({p}_{t})$$

The dual variable associated with this constraint *p*_*t*_ may be interpreted as the emission allowance price that ensures that the representative firm’s strategy coincides with its long-run equilibrium strategy. In other words, presented with these prices, the representative firm does not have an incentive to change its strategy. Note that the supply of allowances ${\overline{q}}_{t}$ is the net supply of emission allowances, corrected for the actions of the MSR.

### Numerical solution strategy

To determine the equilibrium emission allowance price over the studied period (2018–2062) which ensures that the supply of and demand for allowances is balanced (Eq. (6)), we leverage an iterative price-search algorithm, based on ADMM^19^. In each iteration, the algorithm proposes a new emission allowance price *p*_*t*_ for each considered year, depending on the imbalance between the allowances requested by the firms ${\tilde{q}}_{t}$ and the net supply of allowances ${\overline{q}}_{t}$, which in turn depends on the cap and the MSR actions.

After each update of the price and net supply of emission allowances, the representative firm re-optimizes its decisions. If the emission allowance price, MSR actions, hence, net supply of emission allowances, and the representative firm’s strategy no longer change between iterations, we accept the solution as an equilibrium solution.

By adopting this solution strategy, one effectively parameterizes the decision problem of the representative firm in the emission allowance price, which in turn is a function of the net supply and MSR actions. Consequently, the emissions of the representative firm may be calculated ex-ante, facilitating the use of any non-linear marginal abatement cost curve, and the representative firm’s decision problem becomes a linear programming problem, which can be solved efficiently using off-the-shelf solvers. For details on the adopted solution strategy and its convergence, the interested reader is referred to Bruninx et al.^19^.

### Marginal abatement cost curves

Since the marginal abatement cost curve of sectors covered by the EU ETS is fundamentally uncertain, we run each demand shock scenario for a comprehensive set of marginal abatement cost curves, which all adhere to the following functional form:

7 $$\forall t\,\in \,{{{{{{{\mathcal{T}}}}}}}}:\quad {p}_{t}=\beta \cdot {(\overline{E}-{q}_{t})}^{\gamma }$$

In each year *t*, the marginal abatement cost *p*_*t*_ is defined by baseline emissions $\overline{E}$, a slope *β* and a curvature *γ*.

This set of time-invariant marginal abatement cost curves is obtained as follows. Baseline emissions are set to 1900 MtCO_2_, as in Perino and Wilner^28^. The real discount rate is set to 8%. The curvature *γ* is varied between 0.5 and 4.2, with increments of 0.05. For each curvature value, the slope of each abatement cost curve is calibrated to reproduce the average 2019 emission allowance prices (24.7 €/tCO2, based on EEX^29^) while imposing observed emissions in 2019 and the state of the EU ETS at the start of 2019^30,31^. Note that the Green Deal was first announced in December 2019, and hence, is assumed not to be internalized by market parties in the average 2019 prices.

The emission allowance cap in these simulations is in line with 2019 policy, i.e., assuming a −40% emission reduction target by 2030, no negative demand shock due to COVID-19 nor a reduction of the cap as of 2021 to reflect the impact of Brexit on the geographical coverage of EU ETS. The MSR is governed by the 2018 design. This implies that aviation emission allowances and the corresponding cap is not considered by the MSR, hence, for sake of simplicity, these emissions and the aviation emissions cap are excluded during our calibration effort. *γ*-values below 0.5 yield emissions in 2020 that would exceed 2017 levels, which is, at current emission allowance prices, deemed unrealistic. *γ*-values above 4.2 lead to waterbed closures after 2050 in scenario (1). The resulting set of 75 MACCs is then fixed and used to study different policy and demand shock scenarios. Note that we do not aim to quantify which abatement cost curve is more realistic.

### Estimating waterbed leakage (Fig. 1)

To estimate waterbed leakage associated with an overlapping policy (Fig. 1), we take the following approach. From the set of calibrated marginal abatement cost curves (see above), we select a subset that ensures that each year in which the waterbed may be sealed (2023–2050 under the 2018 EU ETS and MSR design or 2023–2038 under the proposed design in the Fit for 55 Package) occurs in the output once. For each of these marginal abatement cost curves, we compute a reference equilibrium emission and EUA price path assuming a −40% (2018 EU ETS and MSR design) or −55% emission reduction target for 2030 (Fit for 55 Package), considering the impact of COVID-19. In a second set of simulations, considering the same marginal abatement cost curves and policy boundary conditions, we add an overlapping policy reducing the demand for emission allowances by 1 MtCO_2_ in a year between 2020 and 2050. Comparing cumulative emissions in the second set of simulations to the corresponding reference result yields an estimate of waterbed leakage. To estimate waterbed leakage of policies on the margin, we ensure that none of the simulated overlapping policies (i) affect the duration of the waterbed puncture, (ii) avoid placing allowances in the MSR due the minimum intake rate of 100 MtCO_2_, unless this is the case without the overlapping policy, (2018 EU ETS and MSR design)^8^ or (iii) change the year in which the TNAC falls below 1096 MtCO_2_ (Fit for 55 Package)^4^. To make this more useful for researchers and policy makers, the numerical values of waterbed leakage for a waterbed sealing in the period 2023–2050 (2018 EU ETS and MSR design) or 2023–2038 (Fit for 55 Package) and overlapping policies in the period 2020–2050 can be found in the Source Data.

To separate the direct and indirect effect that jointly determine the waterbed leakage (Supplementary Figs. 1 and 2), we calculate the direct effect for each of those policies or shocks as follows. We apply the full set of MSR rules to a hypothetical emission profile, in which we add the demand shock directly to the reference equilibrium emission profile (see previous paragraph). This implies—by definition of the direct effect—a one-on-one relation between the change in emission allowance demand and the emission profile. By (i) computing the difference in supply of emission allowances associated with the reference equilibrium emission profile and the hypothetical emission profile considering the overlapping policy and (ii) normalizing this value to the magnitude of the demand shock, we obtain the direct effect.

The direct effect under the 2018 EU ETS and MSR design can also be estimated analytically as follows. For every ton of CO_2_ affected by the overlapping policy or shock in year *t* (*t* ≥ 2018), direct invalidation of allowances approximately equals^6,10^:

8 $$	1-{(1-0.24)}^{n}\cdot {(1-0.12)}^{m}\;\;\,{{\mbox{with}}}\,\;\; n=\max \left[0,\min \left[2022,{t}^{{{\mbox{sealed}}}}\right]-t\right]\\ 	m=\max \left[0,\max \left[2022,{t}^{{{\mbox{sealed}}}}\right]-\max [2022,t]\right]$$

*n* and *m* are the number of years between *t* and the year in which the waterbed is sealed *t*^sealed^ (i.e., when the TNAC is below the 833 MtCO_2_ threshold or the amount to be transferred to the MSR falls below 100 MtCO_2_), with intake rates of 24% (2019–2023) and 12% (after 2023). More precisely, *n* indicates the number of years between *t* and 2022 or *t*^sealed^, whichever comes first. *m* is the number of years after 2022 between the year in which the policy takes place *t* and the year in which the waterbed is sealed *t*^sealed^. For example, consider a policy that reduces emissions in 2020 by one MtCO_2_ and assume only the direct effect is at play. If the waterbed is sealed by 2023, invalidation equals 1 − (1−0.24)^2^ ⋅ (1−0.12)^0^ ≈ 0.42 MtCO_2_. If the waterbed is sealed by 2030, invalidation equals 1 − (1−0.24)^2^ ⋅ (1−0.12)^8^ ≈ 0.79 MtCO_2_. Note that Eq. (8) does not fully accounts for the MSR’s timing, i.e., the transfer of allowances to the MSR in any year depends on the TNAC in the 2 preceding years. In contrast, our approach (see previous paragraph) fully accounts for these effects.

The direct effect under the proposed design of EU ETS and the MSR in the Fit for 55 Package does not only depend on the year in which the waterbed seals, but also on whether and how long the TNAC falls in the range 833 MtCO_2_ to 1096 MtCO_2_. If the TNAC is never in the range 833 MtCO_2_ to 1096 MtCO_2_, the direct invalidation of allowances can be approximated as before, accounting for the adapted definition of *m* and *n*:

9 $$	1-{(1-0.24)}^{n}\cdot {(1-0.12)}^{m}\;\;\,{{\mbox{with}}}\;\;\, n=\max \left[0,\min \left[2029,{t}^{{{\mbox{sealed}}}}\right]-t\right]\\ 	 m=\max \left[0,\max \left[2029,{t}^{{{\mbox{sealed}}}}\right]-\max [2029,t]\right]$$

If the TNAC falls in the range 833 MtCO_2_ to 1096 MtCO_2_ before the waterbed is sealed, Eq. (9) no longer holds. Computing the direct change in invalidation in response to an overlapping policy or shock requires calculating the transfers to the MSR and invalidation on a year-by-year basis, accounting for timing of the impact of the MSR intake on the supply of allowances. Following current practice^31,32^ and assuming the transfers to the MSR are calculated based on the TNAC at the end of year *t* − 1, we subtract 4/12 of allowances to be transferred to the MSR from the supply in year *t* (i.e., September to December) and 8/12 in year *t* + 1 (i.e., January to August). Table 2 illustrates how one may estimate direct waterbed leakage of a shock in 2021, when the TNAC falls in the range 833 MtCO_2_ 1096 MtCO_2_ in 2025 and the waterbed seals in 2027. By enumerating all possible trajectories of the TNAC before the waterbed seals, one can prove the following properties of direct waterbed leakage under the proposed design.

- Direct waterbed leakage is at least 1 if the TNAC falls in the range 833 MtCO_2_ to 1096 MtCO_2_ for at least one, but less than four consecutive years between the moment that the overlapping policy or shock affects emissions and the sealing of the waterbed. Direct waterbed leakage is highest if it affects emission allowance demand in the year before that the TNAC falls in the range 833 MtCO_2_ to 1096 MtCO_2_;
- Direct waterbed leakage equals 1 if the TNAC falls in the range MtCO_2_ to 1096 MtCO_2_ for 1 year before the waterbed seals and the policy or shock affects emissions in that year;
- Direct waterbed leakage is at most 1.67, which occurs when the overlapping policy or shock affects emission allowance demand 2 years before the waterbed seals and falls in the range 833 MtCO_2_ to 1096 MtCO_2_ during those 2 years.

**Table 2:** A policy that decreases the demand for allowances by 1 MtCO_2_ in 2021 decreases cumulative supply of emission allowances by 1.22 MtCO_2_, hence, waterbed leakage is 1.22.

Year *t*	TNAC_*t*_	Δ TNAC_*t*_	Δ Intake_*t*_(TNAC_*t*−1_)	Δ Intake_*t*_(TNAC_*t*−2_)
2021	>1096	1	–	–
2022	>1096	1–0.08 = 0.92	0.24 ⋅ 1 ⋅ 4/12 = 0.08	–
2023	>1096	0.92–0.07–0.16 = 0.69	0.24 ⋅ 0.92 ⋅ 4/12 = 0.07	0.24 ⋅ 1 ⋅ 8/12 = 0.16
2024	>1096	0.48	0.24 ⋅ 0.69 ⋅ 4/12 = 0.05	0.24 ⋅ 0.92 ⋅ 8/12 = 0.15
2025	>833, <1096	0.34	0.24 ⋅ 0.48 ⋅ 4/12 = 0.04	0.24 ⋅ 0.69 ⋅ 8/12 = 0.11
2026	>833, <1096	0.15	0.34 ⋅ 4/12 = 0.11	0.24 ⋅ 0.48 ⋅ 8/12 = 0.08
2027	<833	−0.13	0.15 ⋅ 4/12 = 0.05	0.34 ⋅ 8/12 = 0.22
2028	<833	−0.22	–	0.15 ⋅ 8/12 = 0.10
2029	<833	−0.22	–	–

TNAC_*t*_ indicates the TNAC in year *t*, Δ TNAC_*t*_ the change in TNAC in response to the overlapping policy or shock corrected for the change in MSR intake Δ Intake_*t*_(TNAC_*t*−1_) and Δ Intake_*t*_(TNAC_*t*−2_), which depends on the TNAC in year *t* − 1 and *t* − 2.

Note that the estimates in Fig. 1 pertain to policies affecting the demand for emission allowances in a single year. The total waterbed leakage of a policy or a combination of policies spanning over different years can be calculated as the weighted sum of its effect over time, assuming they do not affect the year in which the waterbed is sealed:

10 $$\frac{{\sum }_{t}{\sum }_{i}W{L}_{t}({t}_{sealed})\cdot {q}_{it}}{{\sum }_{t}{\sum }_{i}| {q}_{it}| }$$

where *q*_*it*_ is the effect (in tons of CO_2_) of policy *i* in year *t* on the total number of allowances in circulation, while *WL*_*t*_(*t*_*sealed*_) is the magnitude waterbed leakage in year *t*, which is a function of the year the waterbed is sealed *t*_*sealed*_.

### Estimating cumulative emissions and emission allowance prices in the five scenarios (Fig. 2)

To estimate the impact of the three exogenous shocks on cumulative emissions and emission allowance prices (Fig. 2), we compute an equilibrium for each of the calibrated marginal abatement cost curves (see above) in five different policy scenarios:

- In scenario (1), we consider the current 2030 EU ETS emission reduction target of −43% relative to 2005 emission levels and do not consider the impact of the COVID-19 pandemic on emission allowance demand. The emission cap and LRF are rebased to reflect the impact of Brexit as of 2021^22^. In addition, we also include aviation in the European Economic Area and assume the aviation emissions allowances and emissions from this sector are fully accounted for in the calculation of the TNAC, as is proposed in the Fit for 55 Package^4^.
- In scenario (2), we add the negative demand shock of the COVID-19 pandemic to scenario (1), modeled as a reduction in baseline emissions. We assume the demand shock linearly decreases from its initial value in 2020, 240 MtCO_2_ (the worst-case estimate in Bruninx and Ovaere^2^ and in line with the increase of the TNAC in 2020^32^), to zero at the end of 2025. The total negative demand shock is, hence, 720 MtCO_2_.
- In scenario (4), we add the negative supply shock of the proposed design changes to EU ETS and the MSR in the Fit for 55 Package^4^ to scenario (2). This implies rebasing the emission allowance cap and linear reduction to include maritime transport and increasing the linear reduction factor to 4.2%. The 24% intake rate is maintained throughout Phase IV and the minimum intake limits are removed. Furthermore, if the TNAC falls in the range of 833 MtCO_2_ to 1096 MtCO_2_, the difference between the TNAC and 833 MtCO_2_ is placed in the MSR. The invalidation threshold is set to 400 MtCO_2_. We assume that the considered linear reduction factor is kept constant after 2030 until the supply is zero.
- In scenario (3) and (5) we add a negative and positive demand shock to scenario (4), reflecting the potential impact of overlapping policies funded by the €750 billion NextGenerationEU recovery stimulus package. Since these policies can affect the demand for emission allowances at different times and in many different ways, we will only simulate two generic scenarios. One that increases demand for allowances by 100 MtCO_2_ per year from 2021 to 2030 (e.g., electric vehicles) (scenario (5)), and one that decreases demand for allowances by 100 MtCO_2_ per year from 2021 to 2030 (e.g., renewables support) (scenario (3)).

By executing this analysis for a wide range of marginal abatement cost curves, we illustrate that the duration of the waterbed puncture, and consequently, cumulative emissions, may change in three ways: (i) expectations about marginal abatement costs, (ii) overlapping policies and exogenous shocks, and (iii) the design of EU ETS itself.

First, expectations about future abatement costs will affect the TNAC today. If firms expect higher abatement costs in the future, i.e., a more convex marginal abatement cost curve, they would likely choose to abate more today and bank the surplus allowances for future use. But because of the invalidation rule, if more allowances are banked today, the duration of the waterbed puncture may increase, more allowances will be invalidated, and the cumulative emissions reductions would be greater. In contrast, if firms expect future abatement costs to below, e.g., because of technological learning^33^, they would likely choose to postpone abatement and bank fewer allowances. With fewer banked allowances, the waterbed could be sealed sooner, fewer allowances would be invalidated, and cumulative emissions reductions would be lower^11^. The design of the invalidation rule thus implies a counter-intuitive, self-reinforcing relation between the future cost of abatement and the invalidation volume, making the invalidation rule more stringent when the cost of compliance is higher—an effect first discussed by Bruninx et al.^19^.This surprising result crucially depends on the assumption that market participants are intertemporally optimizing over the full horizon of the EU ETS. Indeed, in models with a limited horizon^26^ market participants do not consider the challenges further in the future and will abate less, which decreases cumulative invalidation. As a result, the positive correlation between the convexity of the marginal abatement cost curve and invalidation increases the more market participants look further into the future, assuming they face the same marginal abatement cost curve. Although future marginal abatement costs are intrinsically uncertain^25^, there is evidence that the marginal abatement cost curve may be (highly) convex^34^.

Second, any European, national, or local policy that affects the demand for allowances may change the TNAC and the duration of the waterbed puncture (Scenarios (3) and (5)). For example, support for electric vehicles increases the demand, because gasoline (not covered by EU ETS) is substituted by electricity (covered by EU ETS), while support for renewable generation or the forced closure of a coal power plant decreases it. If those policies affect the demand for emission allowances before the waterbed is expected to seal, the first measure may decrease the duration of the waterbed puncture, while the second may increase it: depending on the amount of emissions affected and timing of the policy, the year in which the waterbed is sealed may change. If those policies, however, affect the demand for emission allowances after the waterbed is expected to seal, these effects may be opposite, as the indirect or price effect dominates.

Third, expected changes to the design of EU ETS (Scenarios (2) and (4)), i.e., the supply of allowances, will affect the TNAC today and hence potentially change the duration of the waterbed puncture. For example, increasing the future linear reduction factor may prolong the puncture of the waterbed, because more costly future abatement leads to more abatement now. As a result, the linear reduction factor and cumulative emissions are positively correlated, meaning that decreasing the long-run supply of allowances increases invalidation volumes^19^. Again, this underlines the counter-intuitive, self-reinforcing relation between the linear reduction factor—driving the cost of abatement—and the invalidation volume, making the invalidation rule more stringent when the cost of compliance is higher.

### Reporting summary

Further information on research design is available in the Nature Research Reporting Summary linked to this article.

## Supplementary information

Supplementary Information File

Description of Additional Supplementary Information

Supplementary dataset 1

Reporting Summary

## Data Availability

All data to replicate all figures is available via KU Leuven’s GitLab, https://gitlab.kuleuven.be/UCM/ets-ncc. Source data are provided in the same repository. The same data is available via Zenodo (10.5281/zenodo.4923069).

## Code availability

Computer code to replicate all results is available via KU Leuven’s GitLab, https://gitlab.kuleuven.be/UCM/ets-ncc.
