# Supplementary material for: COVID-19, Green Deal and recovery plan permanently change emissions and prices in EU ETS Phase IV
Source: Nat Commun. 2022 Mar 4;13:1165. doi: 10.1038/s41467-022-28398-2 (PMC8897504; doi:10.1038/s41467-022-28398-2)
Supplement: Supplementary file 1 — Supplementary Information File [file 41467_2022_28398_MOESM1_ESM.pdf]

Supplementary Information File

COVID-19, Green Deal & the recovery plan permanently change emissions and prices in EU ETS Phase IV

Kenneth Bruninx[1,2] and Marten Ovaere*[3,4]

[1]Department of Mechanical Engineering, Faculty of Engineering Science, KU Leuven, Belgium
[2]EnergyVille, Belgium
[3]Department of Economics, Faculty of Economics and Business Administration, Ghent University, Belgium
[4]School of the Environment, Yale University, USA

January 7, 2022

Supplementary figures

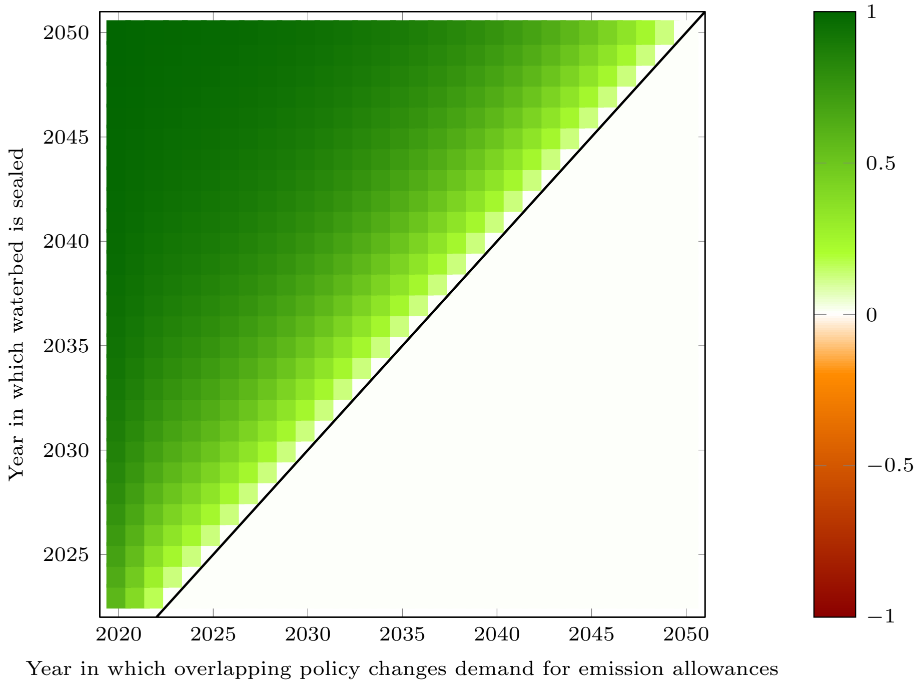

(a) Direct effect

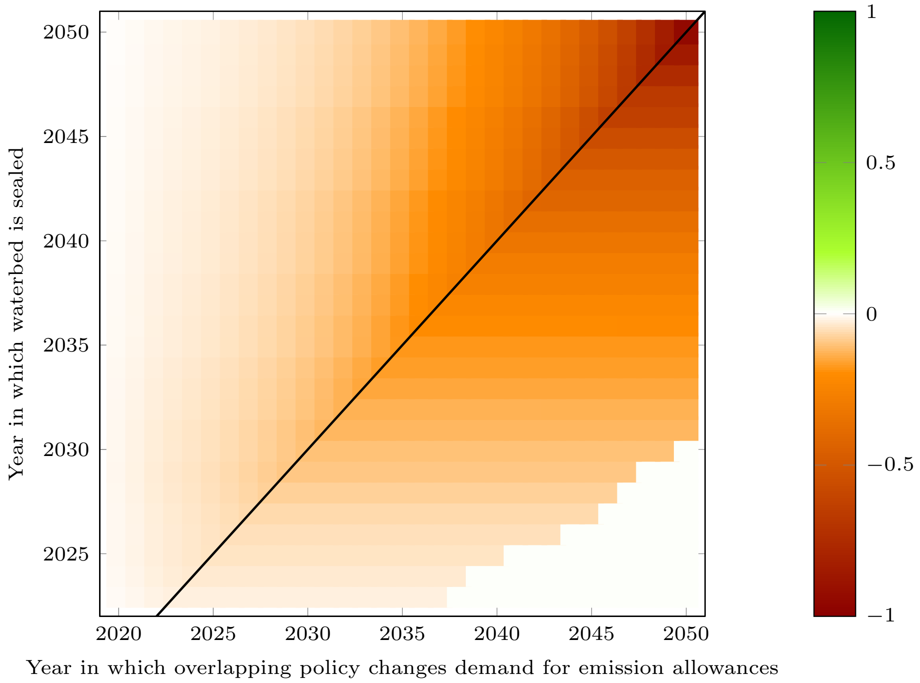

(b) Indirect effect

Supplementary Figure 1. Direct (top) and indirect (bottom) waterbed leakage as a function of the year the overlapping policy or shock takes place (x-axis) and the year the waterbed is sealed (y-axis). The solid black line indicates when the direct effect ends, i.e., a change in emission allowance demand in any year after the waterbed is sealed does not entail a direct effect. Total waterbed leakage can be calculated as the sum of both effects.

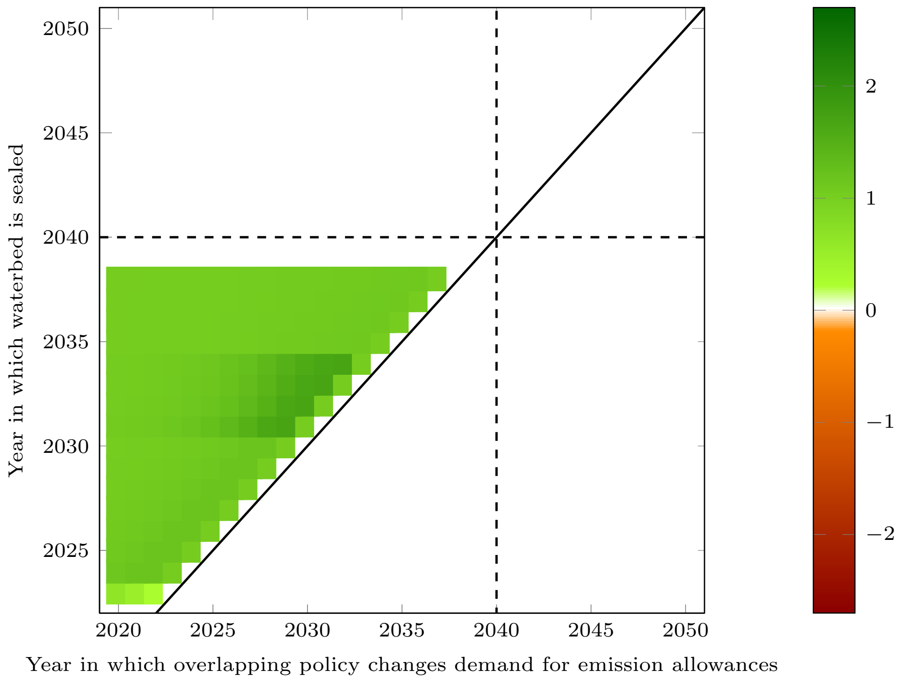

(a) Direct effect

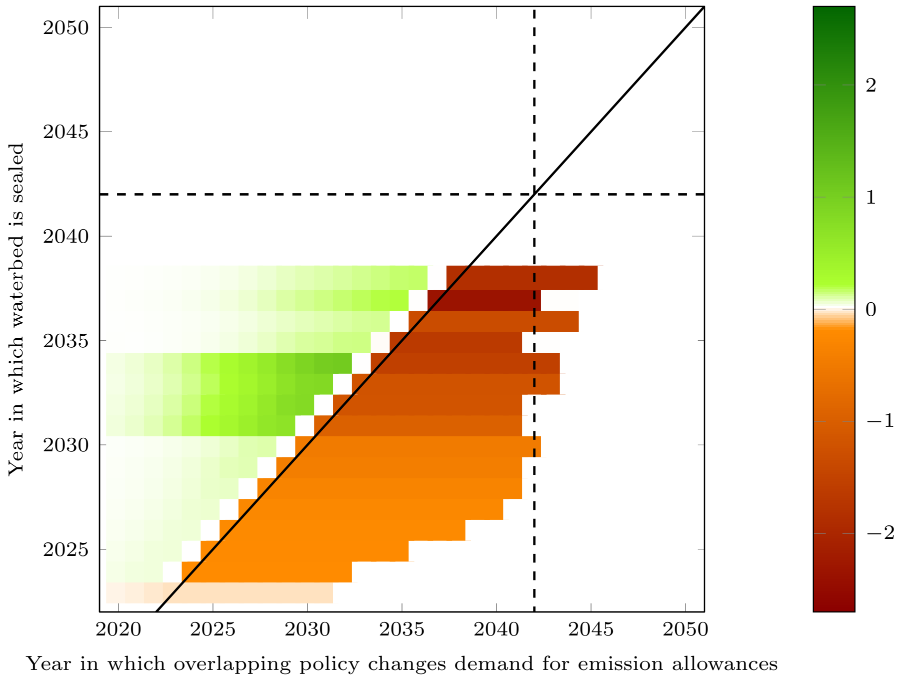

(b) Indirect effect

Supplementary Figure 2. Direct (top) and indirect (bottom) waterbed leakage as a function of the year the overlapping policy or shock takes place (x-axis) and the year the waterbed is sealed (y-axis). The solid black line indicates when the direct effect ends, i.e., a change in emission allowance demand in any year after the waterbed is sealed does not entail a direct effect. Total waterbed leakage can be calculated as the sum of both effects.

Supplementary Table 1. Waterbed leakage of an overlapping policy, depending on the year in which waterbed is sealed (columns) and the year in which the overlapping policy affects the demand for allowances (rows). This table assumes a CO_2 emission reduction target of 40% by 2030 and considers the 2018 EU ETS and MSR design.

Year in which the policy reduces demand for allowances	Year in which waterbed is sealed																											
	2023	2024	2025	2026	2027	2028	2029	2030	2031	2032	2033	2034	2035	2036	2037	2038	2039	2040	2041	2042	2043	2044	2045	2046	2047	2048	2049	2050
2020	0.56	0.61	0.66	0.70	0.74	0.77	0.80	0.82	0.84	0.86	0.88	0.89	0.91	0.92	0.93	0.94	0.94	0.95	0.95	0.96	0.96	0.97	0.97	0.97	0.98	0.98	0.98	0.98
2021	0.37	0.45	0.52	0.58	0.63	0.67	0.71	0.75	0.78	0.81	0.83	0.85	0.87	0.88	0.90	0.91	0.92	0.93	0.93	0.94	0.95	0.95	0.96	0.96	0.97	0.97	0.97	0.97
2022	0.13	0.24	0.33	0.42	0.49	0.55	0.60	0.65	0.69	0.73	0.76	0.79	0.82	0.84	0.86	0.87	0.89	0.90	0.91	0.92	0.93	0.94	0.94	0.95	0.95	0.96	0.96	0.96
2023	-0.03	0.08	0.20	0.30	0.38	0.46	0.52	0.58	0.63	0.68	0.72	0.75	0.78	0.81	0.83	0.85	0.86	0.88	0.89	0.90	0.91	0.92	0.93	0.94	0.94	0.95	0.95	0.96
2024	-0.03	-0.04	0.08	0.19	0.29	0.38	0.45	0.52	0.57	0.63	0.67	0.71	0.75	0.78	0.80	0.82	0.84	0.86	0.87	0.89	0.90	0.91	0.92	0.93	0.93	0.94	0.95	0.95
2025	-0.03	-0.04	-0.05	0.07	0.18	0.28	0.37	0.44	0.51	0.57	0.62	0.67	0.71	0.74	0.77	0.80	0.82	0.84	0.86	0.87	0.89	0.90	0.91	0.92	0.93	0.93	0.94	0.94
2026	-0.03	-0.04	-0.05	-0.06	0.06	0.17	0.27	0.36	0.44	0.51	0.56	0.62	0.66	0.70	0.74	0.77	0.79	0.81	0.83	0.85	0.87	0.88	0.89	0.90	0.91	0.92	0.93	0.93
2027	-0.03	-0.04	-0.05	-0.06	-0.07	0.05	0.16	0.26	0.35	0.43	0.50	0.56	0.61	0.66	0.70	0.73	0.76	0.79	0.81	0.83	0.85	0.86	0.88	0.89	0.90	0.91	0.92	0.92
2028	-0.03	-0.04	-0.05	-0.06	-0.07	-0.08	0.03	0.15	0.25	0.34	0.42	0.49	0.55	0.60	0.65	0.69	0.73	0.75	0.78	0.80	0.82	0.84	0.86	0.87	0.89	0.90	0.90	0.91
2029	-0.03	-0.04	-0.05	-0.06	-0.07	-0.08	-0.10	0.02	0.14	0.24	0.33	0.41	0.49	0.54	0.60	0.64	0.68	0.72	0.75	0.77	0.80	0.82	0.84	0.85	0.87	0.88	0.89	0.90
2030	-0.03	-0.04	-0.05	-0.06	-0.07	-0.08	-0.10	-0.11	0.01	0.13	0.23	0.32	0.41	0.47	0.54	0.59	0.63	0.67	0.71	0.74	0.77	0.79	0.81	0.83	0.85	0.86	0.87	0.88
2031	-0.03	-0.04	-0.05	-0.06	-0.07	-0.08	-0.10	-0.11	-0.13	0.00	0.12	0.22	0.32	0.39	0.46	0.52	0.58	0.62	0.66	0.70	0.73	0.76	0.78	0.80	0.82	0.84	0.85	0.87
2032	-0.03	-0.04	-0.05	-0.06	-0.07	-0.08	-0.10	-0.11	-0.13	-0.14	-0.02	0.10	0.21	0.30	0.38	0.45	0.52	0.57	0.61	0.65	0.69	0.72	0.75	0.77	0.80	0.82	0.83	0.85
2033	-0.03	-0.04	-0.05	-0.06	-0.07	-0.08	-0.10	-0.11	-0.13	-0.14	-0.16	-0.03	0.09	0.20	0.29	0.37	0.44	0.50	0.55	0.60	0.64	0.68	0.71	0.74	0.77	0.79	0.81	0.82
2034	-0.03	-0.04	-0.05	-0.06	-0.07	-0.08	-0.10	-0.11	-0.13	-0.14	-0.16	-0.17	-0.04	0.07	0.18	0.27	0.36	0.42	0.48	0.54	0.59	0.63	0.67	0.70	0.73	0.76	0.78	0.80
2035	-0.03	-0.04	-0.05	-0.06	-0.07	-0.08	-0.10	-0.11	-0.13	-0.14	-0.16	-0.17	-0.18	-0.07	0.06	0.16	0.26	0.34	0.41	0.47	0.53	0.57	0.62	0.65	0.69	0.72	0.74	0.76
2036	-0.03	-0.04	-0.05	-0.06	-0.07	-0.08	-0.10	-0.11	-0.13	-0.14	-0.16	-0.17	-0.18	-0.21	-0.09	0.03	0.15	0.24	0.31	0.38	0.46	0.51	0.56	0.60	0.64	0.67	0.70	0.73
2037	-0.03	-0.04	-0.05	-0.06	-0.07	-0.08	-0.10	-0.11	-0.13	-0.14	-0.16	-0.17	-0.18	-0.21	-0.23	-0.11	0.02	0.12	0.21	0.29	0.37	0.43	0.49	0.54	0.59	0.63	0.66	0.69
2038	0	-0.04	-0.05	-0.06	-0.07	-0.08	-0.10	-0.11	-0.13	-0.14	-0.16	-0.17	-0.18	-0.21	-0.23	-0.27	-0.13	-0.02	0.09	0.18	0.28	0.35	0.41	0.47	0.53	0.57	0.60	0.64
2039	0	0	-0.05	-0.06	-0.07	-0.08	-0.10	-0.11	-0.13	-0.14	-0.16	-0.17	-0.18	-0.21	-0.23	-0.27	-0.29	-0.17	-0.05	0.06	0.17	0.25	0.32	0.38	0.45	0.50	0.54	0.58
2040	0	0	-0.05	-0.06	-0.07	-0.08	-0.10	-0.11	-0.13	-0.14	-0.16	-0.17	-0.18	-0.21	-0.23	-0.27	-0.29	-0.33	-0.21	-0.09	0.04	0.14	0.22	0.29	0.37	0.43	0.47	0.52
2041	0	0	0	-0.06	-0.07	-0.08	-0.10	-0.11	-0.13	-0.14	-0.16	-0.17	-0.18	-0.21	-0.23	-0.27	-0.29	-0.33	-0.37	-0.25	-0.11	0.00	0.10	0.18	0.28	0.34	0.39	0.45
2042	0	0	0	-0.06	-0.07	-0.08	-0.10	-0.11	-0.13	-0.14	-0.16	-0.17	-0.18	-0.21	-0.23	-0.27	-0.29	-0.33	-0.37	-0.42	-0.27	-0.15	-0.04	0.06	0.17	0.24	0.30	0.36
2043	0	0	0	-0.06	-0.07	-0.08	-0.10	-0.11	-0.13	-0.14	-0.16	-0.17	-0.18	-0.21	-0.23	-0.27	-0.29	-0.33	-0.37	-0.42	-0.45	-0.32	-0.19	-0.08	0.04	0.12	0.20	0.27
2044	0	0	0	0	-0.07	-0.08	-0.10	-0.11	-0.13	-0.14	-0.16	-0.17	-0.18	-0.21	-0.23	-0.27	-0.29	-0.33	-0.37	-0.42	-0.45	-0.50	-0.37	-0.25	-0.11	-0.01	0.07	0.15
2045	0	0	0	0	-0.07	-0.08	-0.10	-0.11	-0.13	-0.14	-0.16	-0.17	-0.18	-0.21	-0.23	-0.27	-0.29	-0.33	-0.37	-0.42	-0.45	-0.50	-0.56	-0.44	-0.28	-0.16	-0.07	0.03
2046	0	0	0	0	0	-0.08	-0.10	-0.11	-0.13	-0.14	-0.16	-0.17	-0.18	-0.21	-0.23	-0.27	-0.29	-0.33	-0.37	-0.42	-0.45	-0.50	-0.56	-0.63	-0.47	-0.34	-0.23	-0.12
2047	0	0	0	0	0	0	-0.10	-0.11	-0.13	-0.14	-0.16	-0.17	-0.18	-0.21	-0.23	-0.27	-0.29	-0.33	-0.37	-0.42	-0.45	-0.50	-0.56	-0.63	-0.67	-0.54	-0.42	-0.29
2048	0	0	0	0	0	0	0	-0.11	-0.13	-0.14	-0.16	-0.17	-0.18	-0.21	-0.23	-0.27	-0.29	-0.33	-0.37	-0.42	-0.45	-0.50	-0.56	-0.63	-0.67	-0.75	-0.63	-0.49
2049	0	0	0	0	0	0	0	-0.11	-0.13	-0.14	-0.16	-0.17	-0.18	-0.21	-0.23	-0.27	-0.29	-0.33	-0.37	-0.42	-0.45	-0.50	-0.56	-0.63	-0.67	-0.75	-0.85	-0.72
2050	0	0	0	0	0	0	0	0	-0.13	-0.14	-0.16	-0.17	-0.18	-0.21	-0.23	-0.27	-0.29	-0.33	-0.37	-0.42	-0.45	-0.50	-0.56	-0.63	-0.67	-0.75	-0.85	-0.95

Supplementary Table 2. Waterbed leakage of an overlapping policy, depending on the year in which waterbed is sealed (columns) and the year in which the overlapping policy affects the demand for allowances (rows). This table assumes a CO_2 emission reduction target of 55% by 2030 and considers the EU ETS and MSR design as proposed by the European Commission in the Fit for 55 Package.

Year in which the policy reduces demand for allowances	Year in which waterbed is sealed																											
	2023	2024	2025	2026	2027	2028	2029	2030	2031	2032	2033	2034	2035	2036	2037	2038	2039	2040	2041	2042	2043	2044	2045	2046	2047	2048	2049	2050
2020	0.61	1.13	1.10	1.07	1.05	1.03	1.03	1.02	1.07	1.05	1.04	1.04	1.00	1.00	1.00	1.00												
2021	0.43	1.18	1.14	1.10	1.07	1.05	1.04	1.03	1.10	1.08	1.06	1.06	1.00	1.01	1.01	1.00												
2022	0.20	1.19	1.18	1.14	1.10	1.07	1.05	1.04	1.14	1.11	1.09	1.09	1.01	1.01	1.01	1.01												
2023	-0.05	1.00	1.20	1.18	1.14	1.10	1.08	1.06	1.21	1.16	1.14	1.14	1.01	1.01	1.01	1.01												
2024	-0.05	-0.21	1.00	1.20	1.19	1.15	1.11	1.08	1.30	1.23	1.20	1.20	1.02	1.02	1.02	1.02												
2025	-0.05	-0.21	-0.23	1.00	1.20	1.20	1.16	1.12	1.44	1.34	1.29	1.29	1.03	1.02	1.03	1.02												
2026	-0.05	-0.21	-0.23	-0.24	1.00	1.22	1.21	1.17	1.64	1.49	1.42	1.42	1.04	1.04	1.04	1.03												
2027	-0.05	-0.21	-0.23	-0.24	-0.28	1.00	1.23	1.22	1.90	1.71	1.61	1.61	1.06	1.05	1.06	1.05												
2028	-0.05	-0.21	-0.23	-0.24	-0.28	-0.34	1.00	1.24	2.21	2.00	1.87	1.87	1.09	1.07	1.09	1.07												
2029	-0.05	-0.21	-0.23	-0.24	-0.28	-0.34	-0.45	1.00	2.31	2.35	2.19	2.19	1.12	1.10	1.12	1.09												
2030	-0.05	-0.21	-0.23	-0.24	-0.28	-0.34	-0.45	-0.51	1.00	2.47	2.42	2.44	1.15	1.12	1.15	1.11												
2031	-0.05	-0.21	-0.23	-0.24	-0.28	-0.34	-0.45	-0.51	-0.97	1.00	2.48	2.64	1.17	1.13	1.17	1.13												
2032	0	-0.21	-0.23	-0.24	-0.28	-0.34	-0.45	-0.51	-0.97	-1.20	1.00	2.71	1.19	1.15	1.20	1.15												
2033	0	0	-0.23	-0.24	-0.28	-0.34	-0.45	-0.51	-0.97	-1.20	-1.22	1.00	1.20	1.18	1.23	1.17												
2034	0	0	-0.23	-0.24	-0.28	-0.34	-0.45	-0.51	-0.97	-1.20	-1.22	-1.56	0.99	1.18	1.26	1.19												
2035	0	0	-0.23	-0.24	-0.28	-0.34	-0.45	-0.51	-0.97	-1.20	-1.22	-1.56	-1.66	1.00	1.27	1.22												
2036	0	0	0	-0.24	-0.28	-0.34	-0.45	-0.51	-0.97	-1.20	-1.22	-1.56	-1.66	-1.35	1.00	1.23												
2037	0	0	0	-0.24	-0.28	-0.34	-0.45	-0.51	-0.97	-1.20	-1.22	-1.56	-1.66	-1.35	-2.36	1.00												
2038	0	0	0	-0.24	-0.28	-0.34	-0.45	-0.51	-0.97	-1.20	-1.22	-1.56	-1.66	-1.35	-2.36	-1.86												
2039	0	0	0	0	-0.28	-0.34	-0.45	-0.51	-0.97	-1.20	-1.22	-1.56	-1.66	-1.35	-2.36	-1.86												
2040	0	0	0	0	-0.28	-0.34	-0.45	-0.51	-0.97	-1.20	-1.22	-1.56	-1.66	-1.35	-2.36	-1.86												
2041	0	0	0	0	0	-0.34	-0.45	-0.51	-0.97	-1.20	-1.22	-1.56	-1.66	-1.35	-2.36	-1.86												
2042	0	0	0	0	0	0	0	-0.51	0	0	-1.22	-1.56	0	-1.35	-2.36	-1.86												
2043	0	0	0	0	0	0	0	0	0	0	-1.22	-1.56	0	-1.35	0	-1.86												
2044	0	0	0	0	0	0	0	0	0	0	0	0	0	-1.35	0	-1.86												
2045	0	0	0	0	0	0	0	0	0	0	0	0	0	0	0	-1.86												
2046	0	0	0	0	0	0	0	0	0	0	0	0	0	0	0	0												
2047	0	0	0	0	0	0	0	0	0	0	0	0	0	0	0	0												
2048	0	0	0	0	0	0	0	0	0	0	0	0	0	0	0	0												
2049	0	0	0	0	0	0	0	0	0	0	0	0	0	0	0	0												
2050	0	0	0	0	0	0	0	0	0	0	0	0	0	0	0	0												
