# Supplementary material for: COVID-19, Green Deal and recovery plan permanently change emissions and prices in EU ETS Phase IV
Source: Nat Commun. 2022 Mar 4;13:1165. doi: 10.1038/s41467-022-28398-2 (PMC8897504; doi:10.1038/s41467-022-28398-2)
Supplement: Supplementary file 2 — Description of Additional Supplementary Information [file 41467_2022_28398_MOESM2_ESM.pdf]

Title: Supplementary Data 1

Description: Tables containing the numerical values of Figure 1 and Figure 2.
